# Detection of *Salmonella* spp., *Candida albicans*, *Aspergillus* spp., and Antimicrobial Residues in Raw and Processed Cow Milk from Selected Smallholder Farms of Zimbabwe

**DOI:** 10.1155/2012/301902

**Published:** 2012-09-25

**Authors:** Tryness Anastazia Mhone, Gift Matope, Petronella Tapiwa Saidi

**Affiliations:** ^1^Department of Animal Science, University of Zimbabwe, P.O. Box MP 167, Mount Pleasant, Harare, Zimbabwe; ^2^Department of Paraclinical Veterinary Studies, University of Zimbabwe, P.O. Box MP 167, Mount Pleasant, Harare, Zimbabwe

## Abstract

A cross-sectional study was conducted to detect the presence of *Salmonella* spp., *Candida albicans*, *Aspergillus* spp., and antimicrobial residues in raw milk (*n* = 120) and processed cow milk (*n* = 20) from smallholder dairy farms from three sites in Zimbabwe. Culture and isolation of *Salmonella* spp., *C. albicans*, and *Aspergillus* spp. were performed using selective media, while antimicrobial residues were detected by a dye reduction test. No *Salmonella*, but *C. albicans* (17.5%; 21/120), *Aspergillus* spp. (0.8%; 1/120), and antimicrobial residues (2.5%; 3/120) were detected from raw milk. *C. albicans* was isolated from all three sites, while *Aspergillus* spp. and antimicrobial residues were detected from sites 1 and 3, respectively. From processed milk, only *C. albicans* (5%) was isolated while *Aspergillus* spp. and antimicrobial residues were not detected. These results suggested low prevalence of *Salmonella* spp. and *Aspergillus* spp. and a relatively high prevalence of *C. albicans* in raw milk from the smallholder farms. The potential public health risks of *C. albicans* and the detected antimicrobial residues need to be considered. Thus, educating farmers on improving milking hygiene and storage of milk and establishing programmes for monitoring antimicrobial residues may help to improve the safety of milk from smallholder farms.

## 1. Introduction

Microorganisms such as bacteria and fungi which may gain entry into milk can multiply and bring about spoilage making raw or processed milk unsuitable for human consumption due to rancidity, musty odours, or toxin production [1]. The presence of these microorganisms usually indicates inadequate collection (milking) procedures, poor storage conditions, or unhygienic production [2, 3]. Raw and processed milk can be further rendered unsafe for human consumption by the mere presence of foodborne pathogens such as *Brucella* spp., *Mycobacterium bovis*, *Listeria monocytogenes*, *Campylobacter jejuni*, and *Salmonella* spp. which may not be associated with apparent food spoilage [1].

Contamination of raw milk and products with *Salmonella* spp. is mostly due to infected persons and contamination of the environment, since natural infections of the udder are rare and seldom contribute to human food poisoning. Deficient hygiene in dairies, especially those from developing countries, has often been considered as one of the major reasons for contamination of milk with both spoilage and pathogenic bacteria [2, 4]. Although poultry products are incriminated as the most common sources of nontyphoidal salmonellosis in humans [5], raw milk and milk products are increasingly becoming important sources of human infection [6, 7]. However, foodborne salmonellosis has remained a neglected zoonosis in Zimbabwe and other developing countries, despite an upsurge of cases reported [8]. Hence, the implementation of the “one health medicine” needs to be carefully considered in order to minimise the public health risk of foodborne zoonoses.

The quality of milk also may be affected by the presence of antimicrobial residues that gain access into milk following antimicrobial treatment of mastitic cows [9]. The presence of these residues in milk is undesirable because they may result in hypersensitivity and tissue damage, and antimicrobial resistance in humans [10, 11]. Further, antimicrobial residues may render the milk unfit for processing [12] because they can inhibit microorganisms used in the preparation of processed milk products. Although the regulations governing the dairy industry in Zimbabwe prohibit the sale of antimicrobial residue-adulterated milk for further processing [13], these residues are not closely monitored in smallholder dairies. Therefore, the prevalence of antimicrobial residues and pathogenic microorganisms such as *Salmonella* spp. and fungi in the smallholder dairy sector is not known. Thus, the objective of this study was to investigate the presence of *Salmonella* spp., fungi, and antimicrobial residues in raw and processed milk from selected smallholder dairy farms of Zimbabwe.

## 2. Materials and Methods

### 2.1. Study Sites and Sample Collection

A cross-sectional study was conducted on selected smallholder dairy farms from sites 1, 2, and 3 in Zimbabwe between October 2009 and February 2010. These smallholder dairy sites, operated by the Dairy Development Programme (DDP) with the assistance of the Department of Agricultural Research and Extension (AREX), were selected for this study because they had members who were actively producing raw milk and processed milk products. From each study site, all the farms that were producing and selling milk to the collection centres (depots) were sampled. Duplicate samples of raw milk were collected from individual farms immediately after milking, while processed milk was collected from bulk tanks at milk collection centres. The processed milk (*amasi*) was prepared from milk pasteurised at 80°C and had pH varying from 6.15 to 6.65. Samples were immediately cooled to 4°C and transported to the laboratory for further analysis. Additional information on milking management procedures such as sources of water (closed or open) for use during milking, places of milking, and type of utensils (plastic pails or steel cans) used for collecting and transporting milk to depots were obtained through farmer interviews [14].

### 2.2. Laboratory Tests

The tests for isolation and identification of *Salmonella *spp., *Candida albican*s, and *Aspergillus* spp. and for detection of antimicrobial residues were carried out at Central Veterinary Laboratory and Aglabs (Pvt), in Harare, respectively.

### 2.3. Isolation and Identification of *Salmonella* spp.

The isolation of *Salmonella* spp. was done by enriching samples in Rapport-Vasilliadis (RV) broth (Oxoid) [15]. RV was selected for enrichment based on cost and its reported higher recovery rates for *Salmonella* spp. compared to selenite and tetrathionate broths, even without preenrichment [16]. A sterile swab from raw or processed milk sample was inoculated into RV and incubated at 37°C for 24 hours. A loopful of RV was streaked onto xylose lysine desoxycholate agar (Oxoid) and incubated for a further 24 hours at 37°C.

### 2.4. Isolation and Identification of *Candida albicans *and *Aspergillus* spp.

The isolation of *C. albicans* and *Aspergillus* spp. was carried out on Sabouraud Dextrose agar (Oxoid). One millilitre of the milk samples was inoculated onto culture plates and spread rapidly over the entire agar surface, and plates were incubated at 25°C for up to 7 days. Specific identification of *Aspergillus* spp. and *C. albicans* was as described in detail by Quinn et al. [15]. Briefly, *C. albicans* was identified on the basis of colonial morphology, microscopic appearance of Gram-stained smears, and demonstration of Germ-tube formation. *Aspergillus* spp. were identified based colonial morphology and pigmentation on both the obverse and reverse sides, and by demonstration of characteristic fruiting heads showing conidiophores, vesicles, and conidia in lactophenol cotton blue-stained wet preparations.

### 2.5. Detection of Antimicrobial Residues

The detection of antimicrobial residues (unspecified) in milk was carried out using the dye reduction test that was carried out essentially as described elsewhere [17]. The triphenyl-tetrazolium chloride (TTC) was used as an indicator dye and *Streptococcus thermophilus* as the assay organism. Two 5 mL of milk sample were added to two separate sterile tubes and the volumes made up to 10 mL with antimicrobial-free milk. To one tube, 0.2 mL of a solution of 1000 IU of penicillinase (Calbiochem) per mL was added. To each of the tubes, 1 mL of *Streptococcus thermophilus* of an 18-hour culture was added and thoroughly mixed by inverting the tubes several times. The tubes were incubated in a water bath at 44°C ± 0.5°C for two hours. To each of the tubes, 1 mL of a 1% solution of TTC was added and thoroughly mixed and further incubated at 44°C for 1 hour. The presence of antimicrobial residues in the milk sample was indicated by the development of colourless milk while the control tube turned deep pink due to the reduction of TTC.

## 3. Results and Discussion

This study investigated the presence of *Salmonella* spp., *C. albicans*, *Aspergillus *spp., and antimicrobial residues in the raw and processed milk from selected smallholder dairy farms of Zimbabwe. We were unable to detect *Salmonella* spp. in raw and processed milk samples assessed in this study, even though a high prevalence of faecal *E. coli* was reported in milk samples from these farms [14]. This is the first attempt to isolate *Salmonella* spp. from milk and milk products in Zimbabwe. However, due the small sample size of the milk investigated and the method of detection, the results need to be interpreted with caution. Thus, investigation of more milk samples and attempts to isolate bacteria from lactating dairy cows and from the environmental sources of the dairy farms may provide a better assessment of the potential public health risk of *Salmonella* spp. As reported by Mhone et al. [14], all the farmers in our study practiced hand milking with most of them milking cows in open kraals (makeshift enclosures used for housing cattle), thereby predisposing the raw milk to contamination with *Salmonella *spp. and other bacteria from environmental sources. Although all the farms used aluminium-steel cans for transporting milk, we noted that the use of disinfectants for pre- and postmilking udder disinfection and for disinfecting milking equipment was very erratic with most using water that was obtained from open wells. Since these farmers do not have facilities for cooling milk on-farm, the long delay between milking and transportation of milk to collection centres, which may be far away from the farms, presented an opportunity for growth of spoilage and pathogenic bacteria. In addition, due to the economic depression that has affected Zimbabwe since the year 2000, the supply of electricity to the milk collection centres was regularly interrupted, which is likely to cause further deterioration of the quality of raw milk and milk products [14]. *Salmonella* spp. have been reported in milk samples in some regions in Africa [2] and also in developed countries [18]. The reasons attributed for the positive isolation of *Salmonella* spp. in food include contamination by infected people, contamination from the environment, soil, vegetation, water, and components of animal food such as bone meal, meat meal, fish, or eggs [19, 20]. It has also been noted in some regions that the contamination of milk by *Salmonella* spp. may occur from faecal material of clinically normal cows that are shedding numerous bacteria [21]. The public health risk of *Salmonella* in milk is reduced by pasteurisation and proper hygiene [22]; therefore these precautions are crucial to reduce milkborne infections.

The results of the isolation of *C. albicans* and *Aspergillus* spp. and detection of antimicrobial residues in raw and processed milk investigated in this study are presented in Table 1. The presence of *Aspergillus* spp. in the raw milk samples from site 1 farms could possibly be attributed to contaminated soiled udders and teats, air, and contaminated forage provided to the cows during milking [23]. *Aspergillus* spp. are usually regarded as spoilage microorganisms but may cause invasive disease, aflatoxicosis, and allergic reactions in humans especially those that are immunocompromised and/or on prolonged antibiotic therapy [15, 24].

We further documented the presence of *C. albicans* in both raw and processed milk from the study smallholder dairy farms in Zimbabwe. Although the sources of these *C. albicans* could not be verified based on the available data, it is possible that they could be from mastitic cows [25]. However, milk may be contaminated by infected humans or contaminated environments since *C. albicans* are commensal microorganisms on the mucus membranes of both humans and animals and are also frequently found in the environment [26]. Our results support the observations of others that *C. albicans* are common isolates of raw milk [25]. Considering that these smallholder farmers routinely sell raw milk locally, the public health importance of milkborne *C. albicans* needs to be carefully considered. *C. albicans* is associated with opportunistic infection in both animals and humans, causing “thrush” of the oral cavity and genital tract, especially in immunocompromised people such as those with HIV/AIDS [27], as well as the aged people and young children.

It is noteworthy that antimicrobial residues were detected in raw milk, notwithstanding the small number of samples investigated and the comparative sensitivity of the method employed. Our results support the findings of other studies of developing countries where antimicrobial residues have been detected in milk [12, 28]. These results are likely to reflect failure to observe milk withdrawal periods in cows treated with antimicrobial agents [28], since most of the farmers are usually unaware of the public health risk of these residues [29].

 Although the specific antimicrobial residues were not identified, nor their maximum residue limits specified, their mere presence in milk intended for human consumption was indicative of a potential risk and contravened the local food safety requirements [13]. However, studies which are able to quantify and to detect a broad range of antimicrobial residues other than beta-lactams would give a better magnitude of residue contamination of milk. According to Katzs and Brady [10], consumption of milk containing antimicrobial residues may pose health risks that include allergic reactions such as anaphylaxis, while some may lead to development of aplastic anaemia. Further, these antimicrobial residues may give rise to public health concerns due to the development of antimicrobial resistance in intestinal bacterial populations [11]. These results highlight the need for state veterinary public health authorities to establish monitoring programmes to determine antimicrobial residues in food and set a maximum residual level for these antimicrobial agents.

In conclusion, we were unable to detect *Salmonella* spp. in the raw and processed milk samples assessed in this study. Further studies are required to provide more accurate prevalence rates of *Salmonella* spp. in milk samples from smallholder dairy farms in Zimbabwe. The presence of *Aspergillus *spp., *C. albicans,* and antimicrobial residues in raw milk is a potential public health concern. Thus, educating farmers on improvement of milking hygiene and judicious use of antimicrobials and establishing programmes for monitoring antimicrobial residues may improve the safety of milk from smallholder farms.

## Figures and Tables

**Table 1:** Details of the total numbers and positive milk samples for *Salmonella* spp., *Candida albicans*, *Aspergillus* spp., and antimicrobial residues from selected smallholder dairy farms from sites 1, 2, and 3 of Zimbabwe.

Scheme	Total samples tested	Total positive (%)	Total positive (%)	Total positive (%)	Total positive (%)
		*Salmonella* spp.	*C. albicans*	*Aspergillus* spp.	Antibiotic residues
Site 1					
Raw milk	50	0 (—)	11 (22.0)	1 (2.0)	0
Processed milk	—	—	—	—	—
Site 2					
Raw milk	52	0 (—)	5 (9.6)	0	0
Processed milk	12	0 (—)	1 (8.3)	0	0
Site 3					
Raw milk	18	0 (—)	5 (27.8)	0	3 (16.7)
Processed milk	8	0 (—)	0	0	0

Total					
Raw milk	**120**	0 (—)	**21 (17.5)**	**1 (0.8)**	**3 (2.5)**
Processed milk	**20**	0 (—)	**1 (5.0)**	**0 (0)**	**0 (0)**
